# PTK (protein tyrosine kinase)-6 and HER2 and 4, but not HER1 and 3 predict long-term survival in breast carcinomas

**DOI:** 10.1038/sj.bjc.6603613

**Published:** 2007-02-13

**Authors:** M Aubele, G Auer, A K Walch, A Munro, M J Atkinson, H Braselmann, T Fornander, J M S Bartlett

**Affiliations:** 1 GSF-National Research Center for Environment and Health, Institute of Pathology, D-85764 Neuherberg, Germany; 2 Technical University of Munich, Institute of Pathology, Trogerstr. 18, 81675 München, Germany; 3 Department of Oncology and Pathology, Karolinska Institute and Hospital, S-17176, Stockholm, Sweden; 4 Institute of Pathology, Universitätsklinikum Freiburg, Breisacher Straße115a, 79106 Freiburg I., Br., Germany; 5 Endocrine Cancer Group, Cancer Research Centre, Western General Hospital, Crewe Road, EH4 2XR, South Edinburgh, UK; 6 GSF-National Research Center for Environment and Health, Institute of Molecular Radiation Biology, D-85764, Neuherberg, Germany

**Keywords:** PTK6 (BRK) expression, HER receptors, breast cancer, prognosis

## Abstract

The HER receptors are of therapeutic and prognostic significance in breast cancer, and their function is modulated by cytoplasmic tyrosine kinases like PTK6 (brk). We performed a retrospective study on archival breast cancer samples from patients with long follow-up and compared the protein expression between individual HERs and between HERs and the PTK6. Univariate and multivariate analyses were used to study the prognostic value of parameters. Metastases-free survival of patients for longer than 240 months was inversely associated (*P*⩽0.05) with nodal status, tumour size, and oestrogen receptor status, but was also directly associated with high protein expression levels of HER4 and PTK6 in Kaplan–Meier analysis. In multivariate analysis for metastases-free survival of >240 months, the stepwise selected parameters were tumour size (relative risk 3.1), PTK6 expression (0.4), and number of positive lymph nodes (1.2). Furthermore, we demonstrated a timedependence of the prognostic value attributed to the parameters. The HER receptors (HER2,4), but not PTK6, were independent prognostic markers for metastases-free survival at 60 months, whereas at 240 months PTK6 is the strongest prognostic marker. We demonstrate that PTK6 is a prognostic marker of metastases-free survival in breast cancer, and is independent of the classical morphological and molecular markers of lymph node involvement, tumour size, and HER2 status.

The cytoplasmic non-receptor tyrosine kinase (BRK, PTK6), originally cloned from a human metastatic breast tumour (Mitchell *et al*, 1994), shows elevated expression in breast carcinoma cell lines and in approximately two-thirds of primary breast tumours (Mitchell *et al*, 1994; Barker *et al*, 1997; Llor *et al*, 1999; Born *et al*, 2005). Overexpression of PTK6-mRNA positively correlates with hormone receptor status (Zhao *et al*, 2003) and HER2/NEU expression (Born *et al*, 2005) in breast cancer specimens. Several *in vitro* studies of PTK6 point to a possible role in modulating signal transduction of receptor tyrosine kinases (RTKs): PTK6 expression increases HER3 phosphorylation and Akt activation (Kamalati *et al*, 2000; Zhang *et al*, 2005), sensitises cells to epidermal growth factor (EGF) (Kamalati *et al*, 1996), which activates PTK6 (Kamalati *et al*, 2000; Chen *et al*, 2004), and influences the apoptotic pathway (Haegebarth *et al*, 2005). There are, however, several unanswered questions with regard to the structure, activity, and regulation of PTK6. It was shown by Haegebarth *et al* (2005) that PTK6 negatively regulates the activity of RNA-binding proteins, which may have an impact on the post-transcriptional regulation of gene expression (Haegebarth *et al*, 2005). Furthermore, PTK6 possesses sequences that predicted to form Src homology (SH3 and SH2) domains (Kamalati *et al*, 1996; Zhang *et al*, 2005), suggesting interactions with other proteins. Because of the known involvement of Src in epithelial tumour development and structural similarities with Src, it is thought that PTK6 may play a role in epithelial tumorigenesis (Petro *et al*, 2004; Zhang *et al*, 2005).

The EGF receptor family is composed of four members: EGFR/HER1/erbB1, HER2/NEU/erbB2, HER3/erbB3, and HER4/erbB4 (Mendelsohn *et al*, 2000; Hudelist *et al*, 2003; Lodge *et al*, 2003; Abd El-Rehim *et al*, 2004; Hynes *et al*, 2005). There is a growing body of evidence that members of the HER family are involved in breast cancer development and progression, but the protein expression pattern of all four HER receptors remains poorly understood (Bieche *et al*, 2003; Witton *et al*, 2003; Abd El-Rehim *et al*, 2004; Tovey *et al*, 2005; Bianchi *et al*, 2006). HER1 and HER2 have been consistently associated with a poor prognosis (Witton *et al*, 2003; Abd El-Rehim *et al*, 2004; Wiseman *et al*, 2005), whereas conflicting evidence is available on the prognostic significance of HER3 (Kew *et al*, 2000; Bieche *et al*, 2003; Tovey *et al*, 2004), and HER4 has been more consistently linked to good prognostic factors (PFs) (Witton *et al*, 2003; Abd El-Rehim *et al*, 2004; Tovey *et al*, 2004).

The EGFR-family molecules are outstanding candidate targets for tumour therapy. In breast carcinomas it has been shown that HER2/NEU amplification/overexpression has therapeutic and prognostic implications (Slamon 1990; Meric *et al*, 2002; Hudelist *et al*, 2003; Witton *et al*, 2003; Abd El-Rehim *et al*, 2004; Bianchi *et al*, 2006), and that the monoclonal antibody trastuzumab directed against HER2/NEU is therapeutically active in HER2-positive breast carcinomas (Meric *et al*, 2002; Menard *et al*, 2003). However, a number of HER2/NEU-positive tumours are not responsive to HER2/NEU-directed therapy (Menard *et al*, 2003; Hynes *et al*, 2005), indicating that other HER receptors as well as additional signalling molecules may influence the biological response to trastuzumab.

Ligand binding by HER/erbB receptors induces the formation of receptor homo- or heterodimers and activation of the intrinsic receptor kinase domain, resulting in phosphorylation on specific tyrosine residues (Olayioye *et al*, 2000; Hynes *et al*, 2005) and the creation of binding sites for proteins containing Src homology (SH2-/SH3-) domains (Hudelist *et al*, 2003). The composition of these receptor dimers is thought to influence both quality and quantity of downstream signalling pathways and to determine the cellular response (Olayioye *et al*, 2000; Hudelist *et al*, 2003; Hynes *et al*, 2005). Further, cytoplasmic protein tyrosine kinases containing SH2 or SH3 domains may also modify the RTK signalling (Petro *et al*, 2004; Zhang *et al*, 2005). One such non-receptor protein tyrosine kinase, PTK6 (BRK), is similar to the Src family kinase and includes structural motifs comprising SH2 and SH3 domains.

No study has so far tested the hypothesis that PTK6 may have an impact upon breast cancer prognosis or tested its association with the HER family of RTKs in breast carcinoma tissue. Because of the correlation between PTK6 and HER expression, it is possible that these proteins are together of diagnostic/prognostic relevance. We investigated the protein expression of the HER1–HER4 receptors and of the PTK6 in invasive breast carcinomas with long-term follow-up, and found a previously undescribed time-dependent prognostic relevance of PTK6 using multivariate analysis.

## MATERIALS AND METHODS

### Patients and tumour samples

All studies were performed using formalin-fixed, paraffin-embedded, archival material from invasive breast carcinomas obtained from 193 patients. The patients age ranged from 27 to 84 years (median 66 years), 52 patients were premenopausal, 141 were postmenopausal. The hormone receptor status was evaluated immunohistochemically, revealing that 92 of the tumours were hormone receptor positive. Histological classification (WHO, 2003) defined 168 tumours as being invasive ductal, not otherwise specified. The remaining 25 cases were classified as tubular (7), lobular (9), papillary (3), and medullary (6). A total of 126 cases were classified as grade 2, 48 cases as grade 3, and 19 cases as grade 1, according to Elston and Ellis (1991). Ninety-nine of the tumours were lymph-node-negative, 94 were node-positive, and the majority of the tumours in this study were less than 2 cm in size (*n*=135).

Detailed long-term clinical follow-up was available for 105 patients. These patients do not significantly differ from the full cohort of 193 patients in their clinicohistological parameters. These patients were all surgically treated, and no patient received preoperative treatment. Fifty-four patients did not receive postoperative treatment, whereas adjuvant radio- or chemotherapy was administered to 47 of the patients, and 14 patients received antihormonal treatment. The median clinical follow-up of patients was 144 months (mean 140, range 5–264 months) with 37 patients relapsing with distant metastases and 15 with local recurrence. Ethical approval for the study was obtained from the Ethics Committee of the medical faculty of the Technical University of Munich.

### Tissue microarrays

Tissue Microarray (TMA) construction was performed as described (Richter *et al*, 2000). A haematoxylin- and eosin-stained section from each paraffin block was used to define representative tumour regions. Tissue cylinders of 0.6 mm^2^ in diameter were punched from each paraffin block and transferred to the recipient paraffin block using a tissue-arraying instrument (Beecher Instruments Inc., Silver Spring, MD, USA). From the resulting TMA blocks, 5-*μ*m sections were cut and transferred to adhesive slides using the ‘paraffin-tape-transfer-system’ (Instrumedics, Hackensack, NJ, USA). Both the TMA and punched block were re-examined to validate representative sampling.

### Immunohistochemistry

For immunohistochemistry (IHC), which was performed on all 193 tumours, the following antibodies were used on the TMA sections: HER1 (erbB1/EGFR; 31G7, Invitrogen, Heidelberg, Germany, dilution 1:50); HER2 (erbB2; Hercept kit, K5204, DAKO GmbH, Hamburg, Germany); HER3 (erbB3; H3.105. 5, Stratech, Suffolk, England, 1:20); HER4 (erbB4; H4.77.16, Stratech, 1:20); PTK6 (BRK, C-17, Santa Cruz Biotechnology, Heidelberg, Germany, 1:100).

Deparaffinisation of the TMA sections, antigen retrieval, and incubation of the primary antibody was performed in general as described (Quintanilla-Martinez *et al*, 2003). For PTK6 IHC, the deparaffinised slides were microwave-cooked in 0.01 M citrate buffer (pH 6.0) containing 0.1% Tween 20 at maximum power (800 W) for 8 min. Thereafter, sections were washed in Tris-buffered saline (pH 7.6) containing 5% fetal calf serum (Life Technologies, Grand Island, NY, USA) for 30 min, and the PTK6 antibody was incubated overnight at room temperature. Staining and counterstaining were by an automated immunostainer (Ventana Medical System, Tucson, AZ, USA) (Quintanilla-Martinez *et al*, 2003).

**Figure 1:**
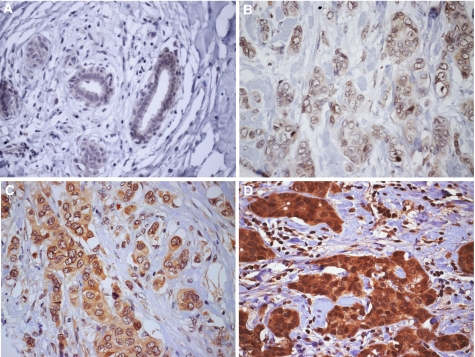
Representative examples of PTK6 IHC (objective magnification × 40) in normal breast epithelium (**A**), and in breast carcinomas classified as 1+ (**B**), 2+ (**C**), and 3+ (**D**).

Tissue staining intensities were scored blind for PTK6, HER3 and HER4 expression by two independent observers using a 4-point scale, where 0=no staining, 1=light staining, 2=moderate staining, and 3=strong staining as published previously (Witton *et al*, 2003). HER1 and HER2 expression were scored using widely accepted criteria that assess the intensity and completeness of membrane staining (Jacobs *et al*, 1999; Ellis *et al*, 2001). Representative examples of the PTK6 expression classification as well as expression in normal ducts are given in Figure 1.

### Statistics

Correlations among the markers, and between markers and clinical parameters, were examined by Spearman's rank correlation test. Survival analysis (for statistical details, see Allison, 1995) was performed using distant recurrence-free survival for follow-up periods of 60, 120, and 240 months, and for disease-free interval of 240 months. The distant recurrence-free survival represents the interval from surgery to the presentation of distant metastases. The disease-free interval was defined as the interval from the date of surgery to the first locoregional recurrence and/or distant metastases.

For univariate survival analysis Kaplan–Meier curves were calculated and differences between strata were tested with the log-rank *χ*^2^ value. Multivariate analysis was performed using Cox proportional hazards regression, using a combined stepwise selection algorithm (SAS Institute, Cary, NC, USA). All parameters reaching a significance level of *P*⩽0.2 in Kaplan–Meier analysis were offered to multivariate analysis. In all other tests, statistical significance was considered at the *P*⩽0.05 level.

For illustration of the multivariate result the parameters, weighted by their coefficients, were used to calculate a new prognostic variable PF (Aubele *et al*, 1995), and patients were grouped according to their PF for Kaplan–Meier analysis.

## RESULTS

### Associations between PTK6 and the HER receptors

Using IHC, strong positivity (2+, 3+) for the HER1 receptor was found in only 5% of the tumours, for HER2 in 26%, for HER3 in 74%, for HER4 in 51%, and for PTK6 in 59% (Table 1). As shown in Table 2, significant correlations were identified among the HER receptors, and between individual HER receptors and PTK6. The strongest correlations (*P*⩽0.0001) were found between HER1 and HER3, between HER2 and PTK6, and between HER4 and PTK6. The histological grade of tumours was significantly associated with HER1, HER4, and with PTK6, and an inverse correlation was identified between the oestrogen receptor (ER) and HER 1 (⩽0.04). No significant association was present between the nodal status or the tumour size and all the immunohistochemical parameters.

To estimate the frequency of HER receptor dimer formation, we calculated the occurrence of combined overexpression of the markers, considering those showing immunohistochemical positivity above the median level as highly overexpressed, whereas a level below the median was considered as low expression (Table 3). High-level co-overexpression of the HER family members and the PTK6 occurred most frequently between HER3 and HER4 (37% of the cases), between HER3 and PTK6 (40% of cases), and between HER4 and PTK6 (34% of the cases), whereas all other combinations were below 18% (Table 3).

### Prognostic relevance of PTK6 and the HER family members

#### Univariate analysis

In univariate analyses lymph node status (*P*=0.001), tumour size (*P*=0.002), and ER status were all inversely correlated with an event-free (distant recurrence) survival of patients longer than 240 months, whereas histological grade and type and the progesterone receptor status were not significant (Table 4).

**Figure 2:**
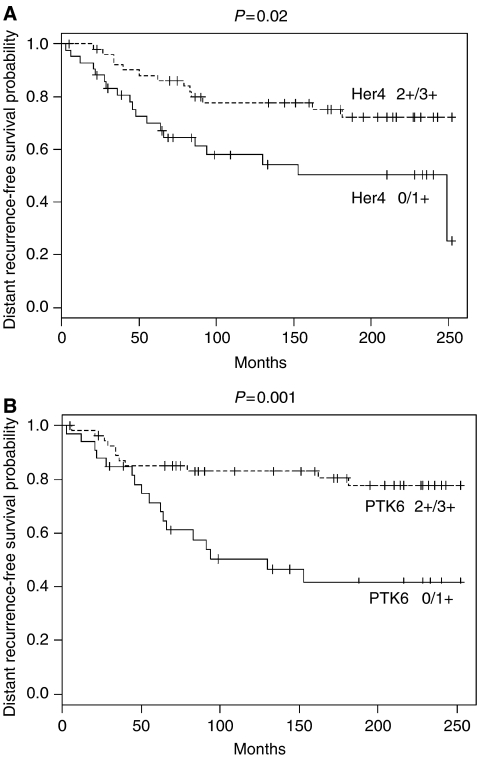
Kaplan–Meier curves for an event-free (distant recurrence) survival of patients of >240 months, with low (0/1+) *vs* high (2+/3+) protein expression of HER4 (**A**) and PTK6 (**B**). The individual groups are: HER4: low=42 patients, 55% remain event-free; high=52 patients, 75% event-free. PTK6: low=33 patients, 48% event-free; high=56 patients, 80% event-free.

A significant positive correlation with the event-free (distant recurrence) survival of patients was identified for HER4 (*P*=0.015) and for PTK6 (*P*=0.001). Figure 2 shows Kaplan–Meier curves demonstrating distant recurrence-free survival of patients with high (2+/3+) *vs* low (0/1+) levels of PTK6 and HER4 expression, respectively.

#### Multivariate prognostic analysis

Multivariate analysis was used to assess the influence of markers on the distant recurrence-free survival of patients, together with that of the clinicopathological parameters. The stepwise selected parameters for a distant recurrence-free survival of patients were tumour size (relative risk 3.1), number of positive lymph nodes (1.2), and PTK6 expression (0.4). The *P*-values, coefficients, relative risk as well as the 95% confidence intervals are summarised in Table 5A.

To study the time dependence of the prognostic value of parameters, we performed multivariate analyses also for 60 months and for 120 months event-free (distant recurrence) survival. The results, which are summarised in Table 5B and C, show a strong time dependence of the prognostic meaning of single parameters. For an interval of 60 months tumour size (relative risk 4.2), HER2 expression (1.7), and the HER4 expression (0.4) were each independently prognostic. At a follow-up of 120 months, the protein expression of HER4 was no longer significant, instead PTK6 expression becomes meaningful. Here, the stepwise selected parameters were tumour size (relative risk 3.4), HER2 (1.5), and PTK6 (0.4). Summarizing these results, the protein expression of HER receptors (HER2, HER4) are of strong prognostic value in short-term survival analysis (60 and 120 months), whereas for a long interval of 240 months the prognostic value of the PTK6 is the strongest parameter in addition to tumour size and lymph node status.

Selecting patients' disease-free survival as the end point of the analysis resulted in similar selected parameters with the same trends: whereas the protein expression of the HER receptors is of significant prognostic value within the first years, and PTK6 has weak prognostic meaning for this interval, the HER receptors are less important and the PTK6 is of high independent prognostic value for longer intervals (120 and 240 months). The stepwise selected parameters for a disease-free survival of patients of >240 months are summarised in Table 5D.

**Figure 3:**
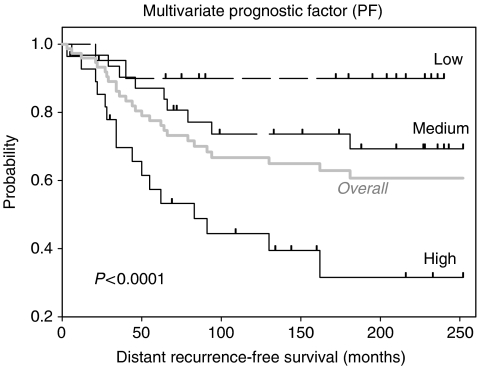
Kaplan–Meier curves for the event-free (distant recurrence) survival of patients of >240 months. Grouping of patients according to their multivariate PF resulted in three different risk groups: low PF (−1.63 to ⩽0.4): 21 patients, 91% remain event-free; medium (0.4 to ⩽1.5): 31 patients, 71% event-free; high PF (1.5–3.24): 28 patients, only 43% event-free. In addition, the overall event-free survival of all patients is plotted in grey.

To illustrate one result of the multivariate analysis, a multivariate PF was calculated for each individual patient by the following linear combination of the variables, weighted by their coefficients (event-free survival 240 months) (Aubele *et al*, 1995): 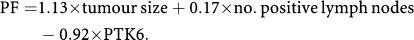


This calculation lead to a continuous variable in a range of −1.63 to 3.24. For Kaplan–Meier analysis, the patients were grouped according to their PF. The resulting curves (Figure 3) demonstrate that 91% of patients with low PF (−1.63 to 0.4) stayed free of distant metastases, whereas from patients with the highest PF (1.5–3.24) only 43% remain metastases-free.

## DISCUSSION

In this study, we investigated the protein expression of the HER receptor family (HER1-HER4) and the cytoplasmic tyrosine kinase PTK6 in archival tissue from 193 breast carcinomas, and tested the association between markers and patient prognosis.

Studies on HER receptors in breast carcinoma so far focused mainly on HER1 and HER2, only some groups have evaluated the expression of the entire HER family (Bieche *et al*, 2003; Hudelist *et al*, 2003; Lodge *et al*, 2003; Witton *et al*, 2003; Abd El-Rehim *et al*, 2004; Wiseman *et al*, 2005; Bianchi *et al*, 2006). Those studies have demonstrated the importance of investigating these related proteins in the context of their ability to cooperatively modify intracellular signalling pathways (Bieche *et al*, 2003; Hudelist *et al*, 2003; Lodge *et al*, 2003; Witton *et al*, 2003; Abd El-Rehim *et al*, 2004; Tovey *et al*, 2005; Wiseman *et al*, 2005, Bianchi *et al*, 2006). We observed in our study strong overexpression 2+/3+ (2+/3+) of the HER receptors rarely for HER1 (5% of tumours), most frequently for HER2 (26%), HER3 (74%), and for HER4 (51% of the tumours), in general in agreement with other studies (Bieche *et al*, 2003; Witton *et al*, 2003; Abd El-Rehim *et al*, 2004). The frequency of combined high overexpression 2+/3+ (2+/3+) of individual HER receptors, which may point to the predominant HER heterodimerization partners in tumours, generally corresponds with other studies (Alimandi *et al*, 1995; Holbro, 2003; Hudelist *et al*, 2003; Witton *et al*, 2003; Abd El-Rehim *et al*, 2004; DiGiovanna *et al*, 2005; Wiseman *et al*, 2005). However, a high co-overexpression of HER3–HER4 in our study was most frequent (37% of tumours) among the HER receptors. We also could not confirm a relationship between HER1–3 and the ER, as was shown by Witton *et al* (2003). Those contrary results may be explained by the different patient cohorts investigated and the low number of ER-positive cases in our cohort.

Overexpression of the HER1 and/or HER2 receptors by breast tumours generally predicts poor patient prognosis (Slamon 1990; Witton *et al*, 2003; Abd El-Rehim *et al*, 2004; Wiseman *et al*, 2005), corresponding to our findings. The prognostic significance of HER3 and HER4 expression in breast carcinoma differs in several reports (Kew *et al*, 2000; Suo *et al*, 2002; Bieche *et al*, 2003; Witton *et al*, 2003; Abd El-Rehim *et al*, 2004; Tovey *et al*, 2004). As, for example, mRNA overexpression of HER3 and HER4 corresponded to a worse prognosis (Bieche *et al*, 2003), whereas combined HER3–HER4 expression showed association with better outcome in another study (Abd El-Rehim *et al*, 2004). Although Wiseman *et al* (2005) did not find a relationship between HER4 expression and patient outcome, in most other studies patients with HER4 overexpression were shown to have increased survival (Suo *et al*, 2002; Witton *et al*, 2003; Abd El-Rehim *et al*, 2004; Tovey *et al*, 2004). The latter is also in good agreement with our findings here, where high HER4 expression shows a direct correlation with the event-free (distant recurrence) survival of patients. Our multivariate results further show independent prognostic values for HER2 and HER4 expression 60 (60 months), and HER2 expression 120 (120 months); however, these results are dependent on the time interval investigated. A similar time dependence in a prognostic marker has been reported by Tovey *et al* (2005). Investigating ER-positive tamoxifen-treated patients, the authors demonstrated a time-dependent predictive value of PR and HER1-3 expression and identified patients at high risk of tamoxifen resistance only in the first 3 years of treatment. This time dependency of the prognostic importance of parameters as well as investigation of different tumour cohorts may explain at least some of the contradictary results in the literature about prognostic value of the HER receptors.

Expression of PTK6 in conjunction with that of the HER receptors has not previously been analysed in breast cancer tissue. We identified a high co-overexpression of PTK6 less frequently with HER1 or HER2 (less than 18%), and more frequent with HER4 (34%) and with HER3 (40% of the tumours). We further found a significant correlation between PTK6 and HER2 at the protein level, confirming our results in a recent mRNA study (Born *et al*, 2005). In the study presented here, strong correlations were also identified between PTK6 and HER3, and HER4, which delivers further support to the theory that PTK6 plays a functional role in the HER signalling network.

PTK6 expression and its cellular consequences in tissues appear paradoxical. In normal breast epithelium, PTK6 is low or undetectable, but the protein is overexpressed in many breast carcinomas (Petro *et al*, 2004; Zhang *et al*, 2005), suggesting, PTK6 expression is related to carcinogenesis. In contrast, high levels of PTK6 are expressed in some differentiating epithelial tissues such as normal gastrointestinal tract, skin (Llor *et al*, 1999; Haegebarth *et al*, 2004), and prostate (Derry *et al*, 2003), and PTK6 expression was associated with the degree of differentiation of breast tissue as indicated by ER expression (Zhao *et al*, 2003). The correlation of PTK6 protein expression with the histological tumour grade observed in our study may also support an association between PTK6 and cell differentiation.

The most striking result in our study is the previously undescribed prognostic relevance of the PTK6 protein expression established using multivariate survival analysis. The time dependence of HER2/HER4/PTK6 expression, relative to survival analysis, may be an important observation. We hypothesise that PTK6 could be a cytoplasmic target influencing the biological response of HER receptor signalling, particularly in the context of HER4 expression. It is well accepted that different HER receptors transduce signals through associations with a variety of cytoplasmic target proteins containing SH2 and/or SH3 domains (Zhang *et al*, 2005). PTK6 possesses both SH2 and SH3 domains (Kamalati *et al*, 1996; Zhang *et al*, 2005), which might enable it to participate in signalling processes. On the basis of our results here, there appears to be an interplay between PTK6 as an indicator and the HER receptors as prognostic markers, in particular HER4. The long-term prognostic value of PTK6, namely that it is the strongest prognostic marker at 240 months but not at 60 months may further support a role for PTK6 in promoting differentiation of tumour cells. Although HER4 expression (low/high) shows prognostic power over the first 5 years of follow-up (Figure 2), survival curves relative to PTK6 expression do not separate during the first 50 months. Longer follow-up, however, suggests a greater prognostic significance for PTK6 expression than HER4. It should be noted that, both markers are strongly correlated and both are directly correlated with favourable clinical outcome, suggesting that they represent competing drivers in multivariate analysis.

To our knowledge, this is the first study describing an independent prognostic value for PTK6 in breast carcinomas, and the association between expression of PTK6 and the HER receptors. These data suggest that PTK6 may have an important role in the signal transduction of HER expressing tumour cells as signalling modifier. Further, our results emphasise not only the need of investigating all four HERs, but also relevant intracellular signalling markers, such as CTK's, which may markedly modify the biological response to extracellular ligands. Kinomic profiling of signalling pathways may provide additional insights to the clinical relevance of key ‘nodes’ in these pathways. The relevance of the time dependence of the markers employed suggests these markers select different subgroups of patients with different prognosis. The combinations of proteomic markers identified here require further investigation in the context of specific treatment regimens in a predictive study large enough to probe their relevance in the context of breast cancer therapy.

## Figures and Tables

**Table 1:** Frequencies of cases (number/percent of cases) scored as 0/1+/2+/3+ according to their expression of HER receptors and PTK6 in primary breast carcinomas

	**HER1/EGFR**	**HER2/neu**	**HER3**	**HER4**	**PTK6**
0	146/**81.1**	55/31.6	1/0.6	11/6.4	7/3.8
1+	25/13.9	74/**42.5**	43/25.1	72/42.1	67/37.0
2+	4/2.2	16/9.2	99/**57.9**	77/**45.0**	75/**41.9**
3+	5/2.8	29/16.7	28/16.4	11/6.4	32/17.7

The highest frequency for each marker is printed bold.

**Table 2:** Protein expression of HER receptors and PTK6 in invasive breast carcinomas

	**HER1**	**HER2**	**HER3**	**HER4**	**PTK6**
HER1		n.s.	0.30	n.s.	n.s.
			<0.0001		
HER2			n.s.	0.16	0.31
				0.044	<0.0001
HER3				0.22	0.16
				0.004	0.047
HER4					0.35
					<0.0001
Grade	0.22	n.s.	n.s.	0.21	0.18
	0.004			0.005	0.019
Nodal status	n.s.	n.s.	n.s.	n.s.	n.s.
Tumour size	n.s.	n.s.	n.s.	n.s.	n.s.
ER	−0.18	n.s.	n.s.	n.s.	n.s.
	0.023				
PrR	n.s.	n.s.	n.s.	n.s.	n.s.

Correlations among and between HER receptors and PTK6 are given, as well as relation of markers to clinicopathological parameters (Spearman correlation coefficients, *P*-values, n.s.=not significant, ER=oestrogen and PrR=progesteron receptor status).

**Table 3:** High-level co-overexpression of HER receptors and PTK6 (number/percent of cases)

	**HER2⩾2**	**HER3⩾2**	**HER4⩾2**	**PTK6⩾2**
HER1>0	9/4.7	29/15.0	20/10.4	22/11.4
HER2⩾2		33/17.1	27/14.0	34/17.6
HER3⩾2			71/**36.8**	77/**39.9**
HER4⩾2				66/34.2

Low or high expression was defined being below or above the median level of the single parameters. The highest frequencies of marker combinations are printed bold.

**Table 4:** Results of Kaplan–Meier analysis of the single parameters for an event-free (distant recurrence) survival of patients of >240 months (*P*-values are given)

**Parameter**	***P*-value**
HER1	0.66
HER2	0.14
HER3	0.19
HER4	0.015^a^
PTK6	0.001^a^
PrR	0.72
ER	0.007
Lymph node status +/−	0.001
Tumour size (<20/>=20 mm)	0.002
Histological grade	0.26
Histological type	0.10

Abbreviations: ER=oestrogen and PrR=progesteron receptor status.

a High expression corresponds to better prognosis.

**Table 5:** Results of the Cox multivariate regression analysis

**Parameter**	***χ* ^2^**	***P*-value**	**Coefficients**	**Relative risk**	**95% confidence interval**
*(A) Total interval (>240 months)*					
tumour size (−10, 10–20, >20 mm)	8.70	0.003	1.13	3.11	1.46-6.61
PTK6 expression	9.56	0.002	−0.92	0.40	0.22–0.71
No. of positive lymph nodes	6.67	0.01	0.17	1.18	1.04–1.35
*(B) Interval 120 months*					
Tumour size (−10, 10–20, >20 mm)	9.40	0.002	1.22	3.39	1.55−7.38
PTK6 expression	10.09	0.002	−0.98	0.38	0.21−0.69
HER2 expression	4.71	0.03	0.42	1.52	1.04−2.21
*(C) Interval 60 months*					
Tumour size ( 10, 10–20, >20 mm)	9.20	0.002	1.44	4.22	1.66–10.68
HER4 expression	5.86	0.02	−1.04	0.35	0.15–0.82
HER2 expression	4.86	0.03	0.50	1.65	1.06–2.58
*(D) Total interval: (>240 months)*					
Tumour size (−10, 10–20, >20 mm)	4.08	0.04	0.68	1.97	1.02–3.80
PTK6 expression	8.99	0.003	−0.79	0.45	0.27–0.76
No. of positive lymph nodes	7.27	0.007	0.17	1.19	1.05–1.34

The summary of stepwise selected parameters is given for an event-free (distant recurrence) survival of >240 months (A), 120 months (B), and 60 months (C), and for a disease-free survival of >240 months (D) (positive coefficients increase, and negative coefficients reduce the risk of an event).
